# Quantification of Climate Warming and Crop Management Impacts on Cotton Phenology

**DOI:** 10.3390/plants6010007

**Published:** 2017-02-10

**Authors:** Shakeel Ahmad, Qaiser Abbas, Ghulam Abbas, Zartash Fatima, Atique-ur-Rehman, Sahrish Naz, Haseeb Younis, Rana Jahanzeb Khan, Wajid Nasim, Muhammad Habib ur Rehman, Ashfaq Ahmad, Ghulam Rasul, Muhammad Azam Khan, Mirza Hasanuzzaman

**Affiliations:** 1 Department of Agronomy, Bahauddin Zakariya University, Multan 60800, Pakistan; qaisermultan@gmail.com (Q.A.); maharabbastarar@gmail.com (G.A.); zartashfatima15@yahoo.com (Z.F.); atiqjugg@gmai.com (A.R.); sahrishnaz.agronomist@gmail.com (S.N.); haseebyounis99@gmail.com (H.Y.); rana_g676@yahoo.com (R.J.K.); 2 Department of Environmental Sciences, COMSATS Institute of Information Technology, Vehari 61100, Pakistan; wajidnasim@ciitvehari.edu.pk; 3 Department of Agronomy, Muhammad Nawaz Shareef Agriculture University, Multan 60800, Pakistan; ranahabib11@gmail.com; 4 US-Pakistan Center for Advanced Studies in Agriculture and Food Security (USPCAS-AFS), University of Agriculture, Faisalabad 38040, Pakistan; aachattha1@yahoo.com; 5 Pakistan Meteorological Department, Islamabad 44000, Pakistan; grmet@yahoo.com; 6 Department of Agriculture, District Chiniot 35400, Pakistan; muhammasazamkhan@yahoo.com; 7 Department of Agronomy, Sher-e-Bangla Agricultural University, Dhaka 1207, Bangladesh; mhzsauag@yahoo.com

**Keywords:** cotton phenology, CSM-CROPGRO-cotton model, climate warming, thermal trend

## Abstract

Understanding the impact of the warming trend on phenological stages and phases of cotton (*Gossypium hirsutum* L.) in central and lower Punjab, Pakistan, may assist in optimizing crop management practices to enhance production. This study determined the influence of the thermal trend on cotton phenology from 1980–2015 in 15 selected locations. The results demonstrated that observed phenological stages including sowing (S), emergence (E), anthesis (A) and physiological maturity (M) occurred earlier by, on average, 5.35, 5.08, 2.87 and 1.12 days decade^−1^, respectively. Phenological phases, sowing anthesis (S-A), anthesis to maturity (A-M) and sowing to maturity (S-M) were reduced by, on average, 2.45, 1.76 and 4.23 days decade^−1^, respectively. Observed sowing, emergence, anthesis and maturity were negatively correlated with air temperature by, on average, −2.03, −1.93, −1.09 and −0.42 days °C^−1^, respectively. Observed sowing-anthesis, anthesis to maturity and sowing-maturity were also negatively correlated with temperature by, on average, −0.94, −0.67 and −1.61 days °C^−1^, respectively. Applying the cropping system model CSM-CROPGRO-Cotton model using a standard variety in all locations indicated that the model-predicted phenology accelerated more due to warming trends than field-observed phenology. However, 30.21% of the harmful influence of the thermal trend was compensated as a result of introducing new cotton cultivars with higher growing degree day (thermal time) requirements. Therefore, new cotton cultivars which have higher thermal times and are high temperature tolerant should be evolved.

## 1. Introduction

Cotton (*Gossypium hirsutum* L.) is known as “White-Gold” and is a most important fiber, oilseed and cash crop in Pakistan. It accounts for 1.5% of the gross domestic product GDP and 7.1% of the agricultural economy of Pakistan. The cotton cultivated area, total production bales and yield ha^−1^ were 2.96 million ha, 13.91 million bales and 802 kg·ha^−1^, respectively [1]. Nevertheless, a study showing the adverse effects of climate warming on the phenological stages and phases of cotton was carried out in Punjab, Pakistan. Cotton growth and development are highly impacted due to biotic as well as abiotic stresses. From these stresses, the elevated mean temperature is the major environmental factor which harmfully reduces cotton yield and quality [2,3,4,5,6,7,8].

Climate change has negatively impacted agricultural production and ecological systems of developed and developing countries, which has been demonstrated in various current worldwide climate change studies [9]. A recent Intergovernmental Panel on Climate Change IPCC report indicated that in mostly Asian countries, the integer of cold days as well as nights has been reduced, while the integer of hot days as well as nights and the heat wave frequency has been increased due to the warming trend since about 1950. The mean temperature at the global level has increased by 0.85 °C since the industrial revolution. The decade of the 2000s was the warmest decade compared previous decades and 2014 was the warmest year compared to past years [9]. Similarly, in Punjab, Pakistan, a warming trend has been indicated for the period of the previous three decades and predominantly in the 2000s [10,11].

Phenological stages as well as phases of any crop are affected by changes in weather conditions and crop management practices, which are comprised of sowing times and variety assortments [12,13,14]. Constant variations in sowing times and the growing of new introduced varieties have made it difficult to measure the long-standing response of the phenology of a crop towards the thermal trend [15,16,17,18,19]. The crops’ growth and developmental rate are accelerating in most climatic conditions due to the increasing warming trend due to climate change. Temperature is the most important factor which influences the crop development rate throughout the whole crop lifecycle and ultimately affects the biological and economical yield [20,21]. Consequently, we must be aware of the phenological response of a crop because of changes in the local mean temperature in order to be capable of developing excellent adaptation strategies, for example better agronomic management practices and improved new introduced cultivars that can compensate for the potential harmful impact of the warming trend [22,23,24]. Crop phenological phases are reduced due to the advancement in the phenological stages due to the warming trend. This negative impact on the crop phenology can be reduced by adopting newly introduced cultivars with higher growing degree day requirements and early sowing dates [24,25,26,27]. The relationship between natural changes in the crop environment, agronomical management practices, and the shifting of cultivars cannot be clarified by statistical models as a result of the perplexing collaboration between hereditary qualities, environment, and management. Nevertheless, the utilization of processed-based crop growth models, for example Decision Support System for Agrotechnology Transfer DSSAT and please define, etc., can clarify some of these multifaceted interactions [28,29,30,31,32,33] by permitting scientists to analyze either the effects of a solitary variable at once or the associations among different factors [34,35].

The purposes of this research were (1) to inspect the observed trends of the stages and phases of cotton phenology from 1980 to 2015 in central and lower Punjab, Pakistan; (2) to correlate the observed phenological stages and calculated phases with temperature trends for the same time period; and (3) to recognize the interrelated impact of warming trends and agronomical management practices on the phenology of the cotton crop.

## 2. Results

### 2.1. Temperature Trend

**Figure 1:**
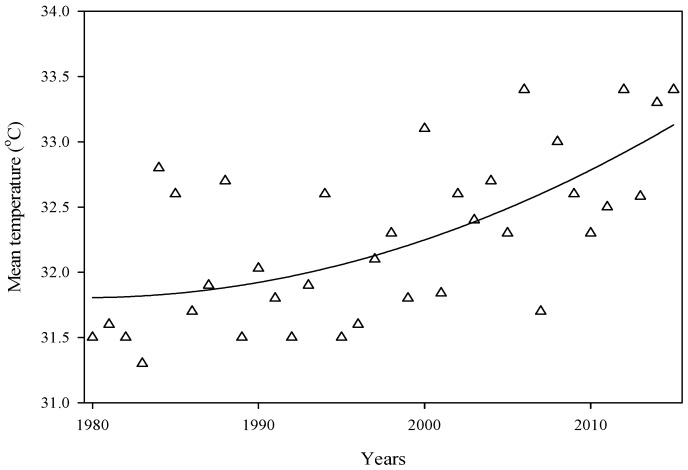
Mean total temperature during the cotton growing season from 1980 to 2015 at 15 sites in Punjab, Pakistan.

**Figure 2:**
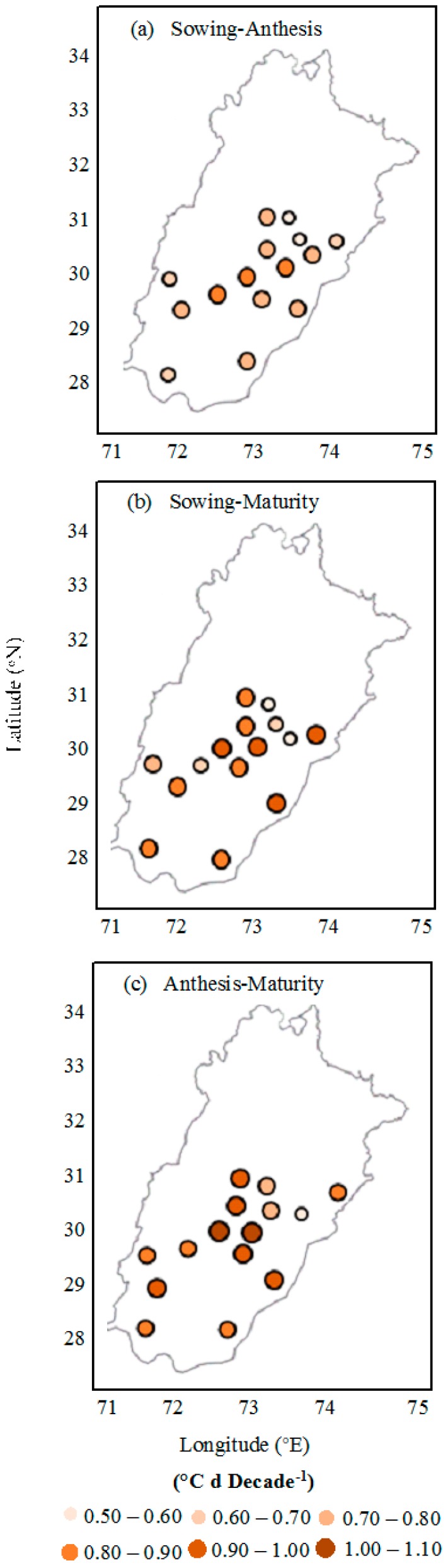
Observed trends in mean temperature during the phenological phases, (**a**) sowing−anthesis, (**b**) sowing−maturity and (**c**) anthesis−maturity, for cotton from 1980 to 2015 at 15 locations in Punjab, Pakistan.

The thermal trend of the environment was observed in the phenological phases of cotton in all 15 chosen locations in central and lower Punjab, Pakistan, for the duration of 1980 to 2015 (Figure 1). The observed air temperature increasing trend all through the phenological phases which included S-A, S-M and A-M ranged from 0.52–0.86, 0.72–1.05 and 0.56–0.99 **°**C decade^−1^, respectively (Figure 2). Overall, the average enhanced temperature values were 0.73, 0.89 and 0.81 **°**C decade^−1^ throughout S-A, S-M and A-M, respectively, in 15 locations in upper and lower Punjab. 

### 2.2. Spatial and Temporal Changes in Phenological Stages of Cotton

**Figure 3:**
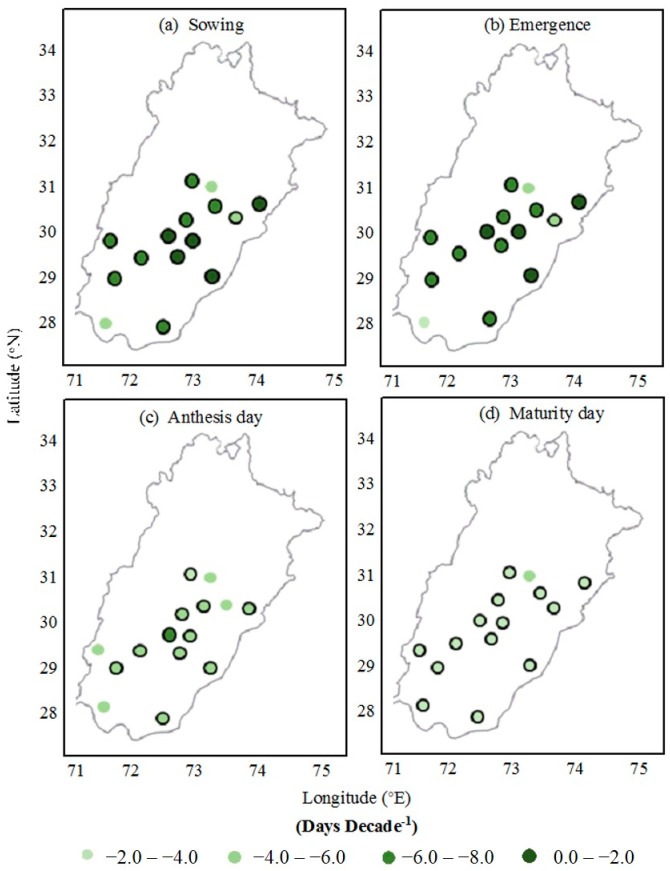
Observed trends in phenological stages of cotton sown from 1980 to 2015 in Punjab, Pakistan; (**a**) sowing; (**b**) emergence; (**c**) anthesis and (**d**) maturity. Circles with black border indicate statistically significant trends at *p =* 0.05 probability level.

Usually, sowing of the cotton crop is done in mid-April to mid-June in central and lower Punjab, Pakistan. The sowing dates of cotton were earlier during 1980 to 2015 in all 15 locations (Table 1). The field-observed range of earliness in the sowing dates of cotton was between 2.80 to 7.40 days decade^−1^ and, on average, the sowing date was 5.35 days decade^−1^ earlier. This earliness of the sowing dates was statistically significant (*p* ˂ 0.05) at 13 locations and non-significant (*p* ˃ 0.05) at two locations. Earliness in emergence dates was observed at all locations and the range of 2.66 to 7.03 days decade^−1^ for emergence dates was observed. However, on average, 5.08 days decade^−1^ were observed in the earliness of the emergence dates (Figure 3). Earliness in the emergence date was significant at 13 locations and non-significant at two locations.

**Figure 4:**
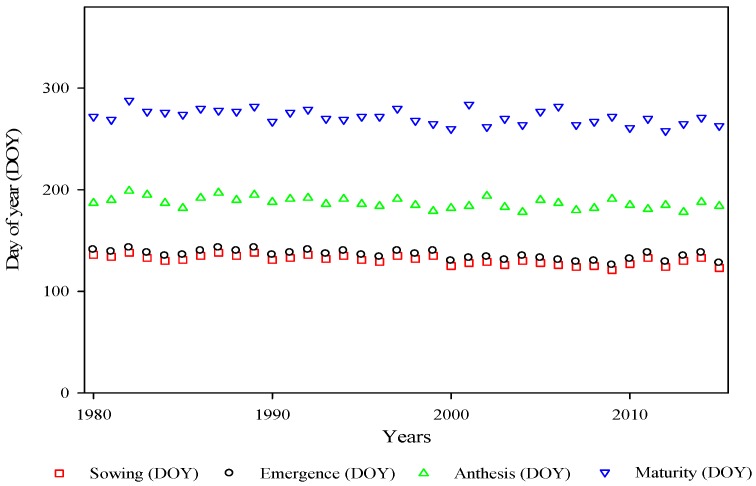
Time series plots of observed dates of onset of phenological stages (sowing, emergence, anthesis and maturity) for cotton from 1980 to 2015 in Punjab, Pakistan.

Anthesis (50% flowering) of cotton in central and lower Punjab, Pakistan, usually starts in June to September (Figure 4). Anthesis dates were advanced, ranging from 1.20 to 4.10 days decade^−1^ at all locations. The average advancement in the anthesis date was −2.87 days decade^−1^. The anthesis date was significant by statistical calculations at 11 locations and non-significant at four locations. The physiological maturity (90% open bolls) date was also advanced at all 15 locations, which ranged from 0.50 to 1.90 days decade^−1^. On average, the cotton physiological maturity date was advanced by 1.12 days decade^−1^. Advancement in the maturity date was significant at 14 locations and non-significant at one location. 

### 2.3. Spatial and Temporal Changes in Phenological Phases of Cotton

**Figure 5:**
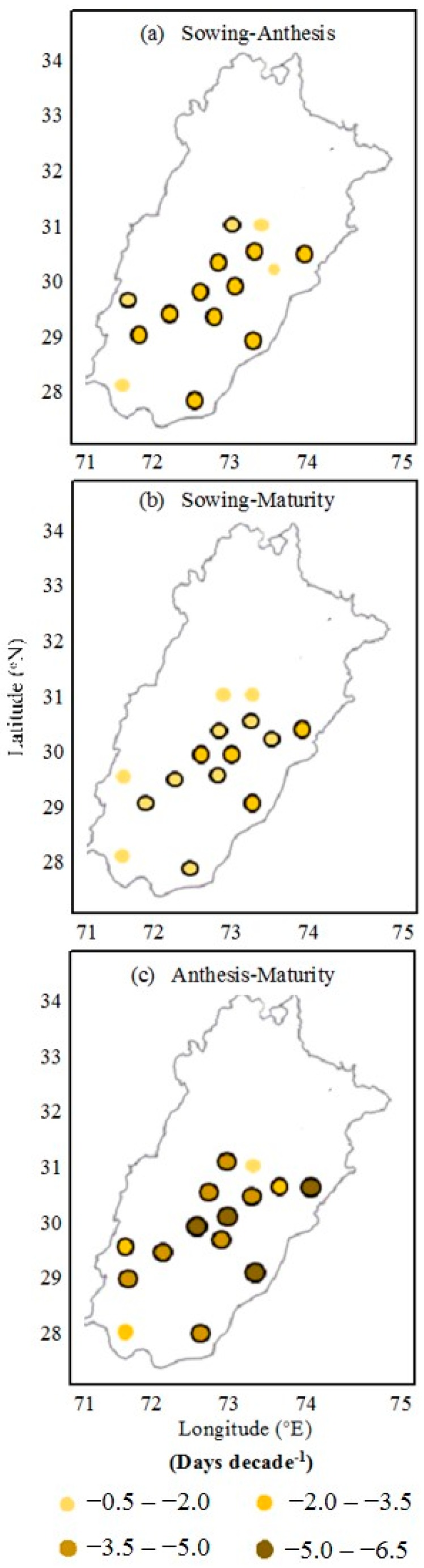
Observed trends in the length of phenological phases for cotton from 1980 to 2015 in Punjab, Pakistan: (**a**) sowing−anthesis; (**b**) anthesis−maturity; and (**c**) sowing−maturity. Circles with black border indicate statistically significant trend at *p =* 0.05 probability level.

The impact of the thermal trend on the cotton crop phenological phases is represented by Figure 5. The cotton crop duration from sowing to physiological maturity was reduced, ranging from 2.30 to 5.66 days decade^−1^ due to the earliness of sowing and physiological maturity. On average, the sowing-to-maturity phenological phase was reduced by 4.23 days decade^−1^. The reduction of the growth duration was statistically significant at 13 locations and non-significant at two locations. The sowing-to-anthesis phase was decreased from the range of 1.02 to 3.30 days decade^−1^ at the locations, which was statistically significant at 12 locations and non-significant at three locations. On average, the reduction in the sowing-to-anthesis phase was 2.45 days decade^−1^. The anthesis-to–physiological maturity phase was decreased 1.76 days decade^−1^ on average, ranging from 0.70 to 2.36 days decade^−1^, which was statistically significant at 11 locations and non-significant at four locations. 

### 2.4. Spatial and Temporal Changes in Thermal Characteristics for Cotton Cultivars

**Figure 6:**
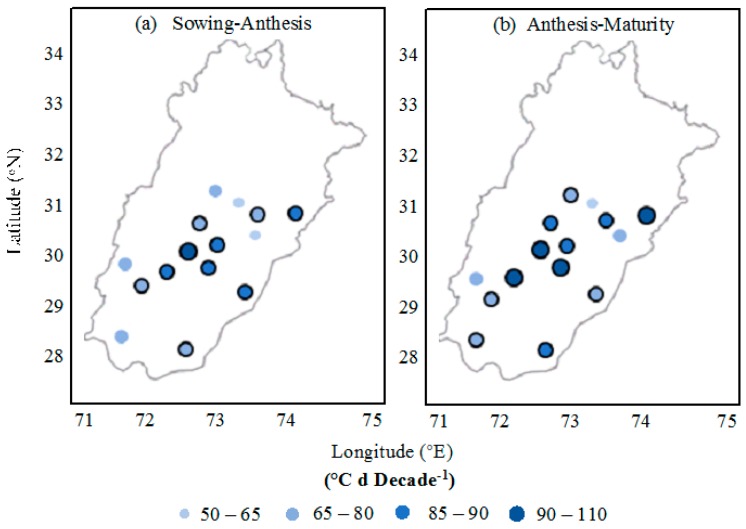
Observed trends in thermal time required for cotton in Punjab, Pakistan, to advance from (**a**) sowing−anthesis and (**b**) anthesis−maturity. Circles with black border indicate statistically significant trend at *p =* 0.05 probability level.

For the cotton cultivars, the total growing degree day requirement from the sowing to anthesis phase was enhanced at all 15 locations, ranging from 53 to 95 °C d decade^−1^ with an average value of 76 °C d decade^−1^. However, this increase was significant at 10 locations and non-significant at five locations (Figure 6). Similarly, the thermal time requisite for the phenological phase of anthesis to physiological maturity was also increased, ranging from 66 to 102 °C d decade^−1^ and 85 °C d decade^−1^ on average. This total thermal time requirement enhancement was significant by statistical analysis at 12 locations and non-significant at three locations.

### 2.5. Correlation of Observed Phenology to Air Temperature

**Figure 7:**
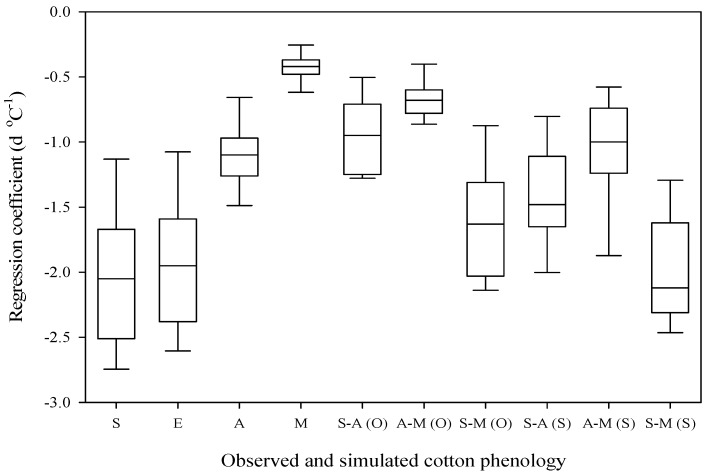
Observed phenology (stages and phases) and simulated phenology (phases) versus temperature trends of cotton, based on data from 15 locations in Punjab, Pakistan, from 1980 to 2015. S: sowing; E: emergence; A: anthesis; M: maturity; S-A: sowing−anthesis; A-M: anthesis−maturity; S−M: sowing−maturity; (O): observed; (S) simulated. Central horizontal line: median; box limits: 25th and 75th percentiles; whiskers: minimum and maximum values.

The regression coefficient of the field-observed phenological stages and phases with the temperature is shown in Table 2 and Figure 7. Negative relationships of sowing dates were obtained with the air temperature at all 15 locations, which were, on average, −2.03 days °C^−1^ and ranged from −1.06 to −2.81 days °C^−1^ (significant at 13 and non-significant at two locations). Emergence dates were also negatively correlated with the thermal trend, which ranged in value from −1.01 to 2.67 days °C^−1^ (significant at 13 and non-significant at two locations) and were −1.93 days °C^−1^ on average. The negative correlation of anthesis with the temperature was obtained, ranging from −0.46 to −1.56 days °C^−1^ and, on average, −1.09 days °C^−1^, which was significant and non-significant by statistical analysis at 11 and four locations, respectively. Physiological maturity was also negatively correlated with the air temperature by an average of −0.42 days °C^−1^ and advanced in the range of −0.19 to −0.72 days °C^−1^ (significant at 14 and non-significant at one location). A negative correlation of the sowing to anthesis phenological phase with the temperature was obtained in the range of −0.39 to −1.32 days °C^−1^ (significant at 12 and non-significant at three locations) and, on average, −0.94 days °C^−1^. Advancement of the sowing to the physiological maturity phase was done by, on average, −1.61 days °C^−1^ and ranged from −0.87 to −2.15 days °C^−1^, which was at 13 locations and non-significant at two locations. A negative correlation of the anthesis to maturity phase with the warming trend was observed, which ranged from −0.27 to −0.90 days °C^−1^ (significant at 11 locations and non-significant at four locations) and was −0.67 days °C^−1^ on average. 

### 2.6. Correlation of Simulated Phenology to Air Temperature

Negative relationships among the CSM-CROPGRO-Cotton model simulated three phenological phases and the warming trend was acquired, which is represented by Table 2 and Figure 7. The sowing-to-anthesis phase was advanced by a mean of −1.42 days °C^−1^ and the values ranged from −0.75 to −2.08 days °C^−1^, which was statistically significant at 14 locations. Advancement of the anthesis to the physiological maturity phase ranged from −0.50 to −2.25 days °C^−1^, which was significant at 13 locations and non-significant at two locations, with −1.06 days °C^−1^ on average. The cotton duration (sowing-to–physiological maturity phase) was shortened by an average of −1.97 days °C^−1^ and the values ranged from −1.21 to −2.50 days °C^−1^ (statistically significant at 14 locations and non-significant at one location).

### 2.7. Model-Predicted and Observed Phenology

The relationship of the correlation of the observed and simulated cotton phenological phases with the air temperature is revealed in Table 3. Temperature sensitivity was higher for the model-predicted phenological phases in contrast to field-recorded phenological phase values. Differences among the model-simulated and field-observed recorded data for S-A, S-M and A-M were 0.48, 0.36 and 0.39 days °C^−1^, respectively, which was statistically significant. The difference in the model-predicted and observed data regarding the cotton phenological phases indicated that the various latest approved/recommended cotton varieties grown by farmers during 1980 to 2015 have higher degree days requirements.

## 3. Discussion

Climate warming had an enormous influence on the phenology of agricultural crops in several regions. The field-recorded changes in the cotton phenological stages and phases in central and lower Punjab, Pakistan, between 1980 and 2015 were possibly caused by the increase in temperature. Nevertheless, other integrated crop management decisions were the changeability in sowing times and the assortment of newly introduced cultivars [36,37] which had a superior total thermal time (total growing degree days) requirement. The latest introduced cultivars and sowing times are, in general, determined through the local agriculturist community, whose preferences regarding varieties and sowing times are adapted in response to the climatic warming tendency in central and lower Punjab, Pakistan. Nonetheless, the precipitation and residual soil moisture for the period of the crop-growing months could also impact the sowing time assessment along with the nutrient uptake by the crops in this region. The earliness of the emergence, anthesis, and maturity (picking) times was due to the earliness of the cotton sowing time. However, certain phenological trends were a result of the distinction in observed thermal trends. On average, the earliness in the sowing and emergence dates of cotton was 5.35 and 5.08 days decade^−1^, respectively, while the advancement of the anthesis and maturity dates was 2.87 and 1.12 days decade^−1^, respectively.

Plant breeding and genetics scientists continuously develop new heat-tolerant cultivars through various breeding techniques that are introduced to the local farmer community subsequent to extensive evaluation [25,38,39,40]. Frequently, these newly cultivars are adapted for local core and non-core cotton area growing conditions, including observed thermal trends. Researchers also have reported innovative phenological characteristics (heat tolerant and climate smart) in improved cotton cultivars [26,35]. In this investigation, the researchers determined the separate influence of the growth of newly introduced cultivars for the 15 selected locations from the field-observed changes through the simulation of the phenology of cotton by means of the same cultivar for the interval of the research period. This crop growth model simulation allowed for the separation of the relations of the temperature and the improved heat-tolerant varietal response [41]. The heat sensitivity of the model simulations was superior as compares to the field-observed phenological data, demonstrating that about 30.21% (Table 3) of the direct negative influence of the warming tendency was mitigated with the evolution of new cotton cultivars that require more photo-thermal time to achieve the various phenological stages in an optimal time [2]. An analogous trend of introducing the latest cultivars which are adapted to a thermal trend was established for other agricultural crops around the world, for example maize in various regions of the USA, and rice and winter wheat in China [35,42]. If the whole duration of the lifecycle of crop varieties is shorter, then as a result, there is a lessening of crop biological and economical yield as a consequence of the lesser time for total dry matter accumulation for the duration of the vegetative segment, chiefly for the highest-input agricultural crops [2,43,44]. As a consequence, the agricultural community chooses newly introduced cultivars, which have higher thermal time requirements for the compensation of the negative effect of the thermal trend on the cotton phenology [35,45].

Crop physiological growth procedures are impacted by temperature, which has a direct impact on the length of the phenological phases and, consequently, affects the ultimate biological and economic yield of cotton [4,5,6,8,46,47]. Whole dry matter accumulation is decreased with the earliness of the anthesis and physiological maturity time due to a mean temperature increasing trend, as a result reducing the crop yield [21]. In numerous regions of the globe, the yield of an assortment of crops has been improved due to a longer reproductive phase (A-M) caused by earliness in flowering and a delayed physiological maturity time [4,42,48,49]. 

Day and night mean temperatures are forecasted to increase in the future as compared to the precedent climatic situation [9]. At the ending of the 21st century, the average temperature is predicted to rise by 2 to 3 °C on the basis of Representative Concentration Pathways RCPs scenarios in Punjab, Pakistan [23]. Moreover, relentless events, for instance heat strokes due to extreme temperature, extreme rainfalls, floods and droughts, are forecasted to be more frequent as well, also potentially negatively impacting agricultural production [50,51]. The phenological crop phases possibly will be potentially reduced for the period of the upcoming decades if the warming trends persist, whereas, at the same time, in temperate and semi-arid regions with high mean temperatures there is also the potential for crop durations to increase if the temperatures are above the cardinal temperatures [24,34,52,53]. As a consequence, the introduction and growth of new heat-resistant cultivars with higher growing degree day (total thermal time) requirements, high temperature tolerance, as well as sowing time adjustments are imperative for mitigation of the harmful impacts of climate change in Punjab, Pakistan [19], and other states across the globe.

## 4. Materials and Methods

### 4.1. Description of Site, Weather, Cotton Management and Phenological Data

**Figure 8:**
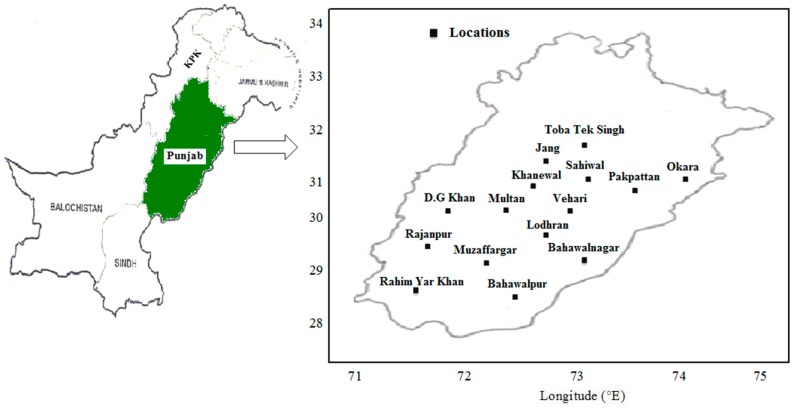
Map showing study locations in Punjab, Pakistan.

Fifteen locations in the conventional cotton zone in Punjab (Figure 8), Pakistan were selected for this study along with 36 years observed weather data were collected from 1980 to 2015 from Pakistan Meteorological Department (PMD), Islamabad. The cotton phenological stages data comprising of sowing, emergence, anthesis (50% flowering) and maturity (90% bolls open) dates were collected from extension wing of Department of Agriculture, Government of Punjab, Pakistan for 15 selected locations. With the assist of these recorded observed phenological stages data, three phenological phases data of cotton crop including S-A, A-M and S-M were determined. Cotton crop management practices were decided by the farming community, and varieties being grown by the framers of selected locations are presented in Table 4. While, other management practices that vary among the farming community; included sowing dates, seed rates, sowing methods, fertilizer amounts, types and application dates, irrigation regimes, methods and application dates, etc. The new cotton variety was being grown by the farming community subsequent to each six to eight years (on an average nine varieties per selected location) and variety selection criteria was higher growing degree day’s requirement. The newly evolved varieties by the breeders were grown by the local farmers on the advice of extension workers. 

### 4.2. Analysis of Observed Data

Relationship between observed phenological stages and phases’ data of cotton crop with average temperature was determined with the help of linear regression equation, in which year was used as independent variable. With the assist of maximum phenological stages at each location, windows for warming trends were gained. For instance, time window for any phenological phase like S-M was from the earlier sowing date to the most recent physiological maturity for the period of last years at each location. In this procedure, the measured warming trend was independent from the corresponded phenological stages and phases variations. 

Correlation of phenological with average temperature of month for the period of month of happening of phenological stages was determined to assess that whether mean monthly temperature influence on phenological stages. Subsequent linear regression equation was used to determine the influence of temperature on cotton crop phenology:

(1) $${\mathrm{OP}}_{\mathrm{nt}}\;={\;a}_{\mathrm{nt}}T_{\mathrm{nt}}+{\;b}_{\mathrm{nt}}\;+\;\varepsilon_{\mathrm{nt}}$$

In Equation (1), observed phenological phases (days) or stages (DOY; day of year) are represented by ‘OP_nt_’ for corresponding nth agricultural weather station or location in respective *t* year. ‘T_nt_’ represents to average daily temperature in centigrade during the relevant phenological stage or phase for particular stations in t year. Regression coefficient (days °C^−1^) of phenological stage or phase responding to temperature is represented by ‘a_nt_’ variable. Variables ‘b_nt_’ and ‘∈_nt_’ stand for intercept and error term for each particular station, respectively. Cotton phenological stages and phases responding to temperature and agronomic management variations are considered by regression coefficient (a_nt_).

### 4.3. CSM-CROPGRO-Cotton Model Descriptions

CSM-CROPGRO-Cotton model was used to isolate outcome of cultivar, management and temperature from the climate warming on phenology changes in cotton phenological stages and phases in fifteen locations. The observed data were compared to simulated data sets. CSM-CROPGRO-Cotton model was calibrated and validated for fifteen locations. Thus total fifteen varieties were calibrated and evaluated. The most commonly grown variety for in a particular location was identified and used for model simulations. After that 1980 to 1982 observed data were used for model calibration and 1983 to 1985 for model validation. Then validated model was applied to simulate the A and M dates for individual location using historical weather data for 1980–2015. The CSM-CROPGRO-Cotton model needed following files for simulation runs; (1) weather file (FILE.WTH) with daily solar radiation, maximum air temperature, minimum air temperature, and precipitation; (2) soil file having soil physical and chemical properties; (3) crop management file and (4) genetic coefficients file. DSSAT version 4.6 was used for the purpose of this research study [54,55]. The detailed information can be found in earlier publications [19,56].

### 4.4. Phenology Simulation with CSM-CROPGRO-Cotton Model and Calculation of Growing Degree Days

The CSM-CROPGRO-Cotton model was employed for simulation of cotton phenology (stages and phases) based on cumulative degree days (GDDs) and crop variety particular GDDs demand for every developmental stage. Photoperiod sensitivity effect the phenology before anthesis. Total accumulation of growing degree days for phenological phases S-A and A-M is measured by following formula:

(2) $$\mathrm{ATT}=\sum_{i\;=\;1}^{n}\mathrm{DTT}$$

In this equation, accumulated thermal time (ATT) per day is represented by daily thermal time (DTT) and n represents to number of days of crop phenology. 

With the help of only one cultivar and keeping similar crop management practices during the years, crop phenological stages and phases were simulated to determine the separate influence of agronomic management practices, variety and temperature on crop phenology. Crop phenology such as anthesis, physiological maturity dates and sowing to maturity phase were predicted from 1980 to 2015 in all selected fifteen locations in upper and lower Punjab, Pakistan. Only one, mostly grown cultivar during 1980–1982 at every location was used for the purpose of calibration of CSM-CROPGRO-Cotton model. Thus, for overall calibration of CSM-CROPGRO-Cotton model at various 15 selected locations, total 15 cultivars were used. After this, crop phenological observed data from 1983 to 1985 was used for the purpose of validation of DSSAT CSM-CROPGRO-Cotton model. Then, crop model was employed to predict crop phenological stages and phases during 1980 to 2015 with the help of same variety and agronomic management practices at every year. Influence of temperature on simulated crop phenological phases such as S-A, A-M, and S-M was determined by linear regression analysis:

(3) $${\mathrm{SP}}_{\mathrm{nt}}=C_{\mathrm{nt}}T_{\mathrm{nt}}+d_{\mathrm{nt}}\;+\;\varepsilon_{\mathrm{nt}}$$

In Equation (3), simulated phenological phases (days) or an event (day of year) is represented by ‘SP_nt_’ for corresponding nth agricultural weather station or location in respective t year. ‘T_nt_’ represents to average daily phenological phase temperature in centigrade during the relevant phenological phase for particular stations in t year. Regression coefficient (days °C^−1^) of phenological phase responding to temperature is represented by ‘c_nt_’ variable. Variables ‘d_nt_’ and ‘∈_nt_’ stand for intercept and error term for each particular location, respectively. Cotton predicted phenological phases responding to temperature and agronomic management variations are considered by regression coefficient (c_nt_). Crop management data including; varieties, sowing dates, seed rates, amount and time of irrigation and method of application, amount and time of fertilizer, organic amendment, tillage and chemical applications commonly used by farming community of each location. Historical weather data and soil physico-chemical properties data were obtained from each location. All data about crop management, weather and soil data were input in CSM-CROPGRO-cotton model for calibration, evaluation and its application.

**Figure 9:**
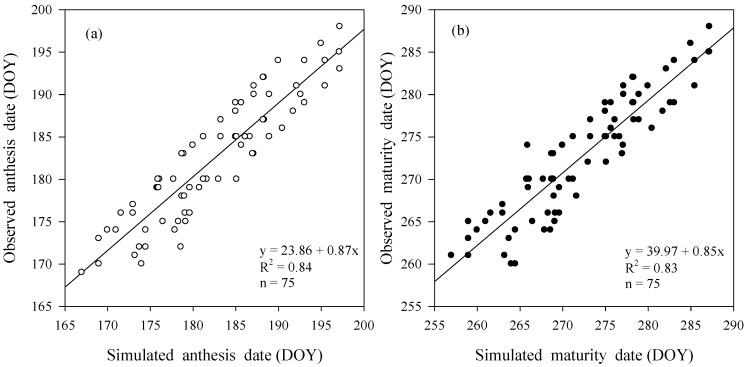
Observed and simulated dates of onset of phenological stages anthesis (**a**; unfilled symbol; left) and maturity (**b**; filled symbols; right) for cotton in Punjab, Pakistan, during 1983−1985; DOY; day of year.

Comparison between models predicted and observed crop phenological stages such as anthesis and physiological maturity dates in years (1983–1985) of model validation at 15 locations is shown in Figure 9. The performance of CSM-CROPGRO-Cotton model was good as model simulated anthesis and physiological maturity dates well matched with observed phenology data (slope = 0.87, *R*^2^ = 0.84, *p* ˂ 0.01) and (slope = 0.85, *R*^2^ = 0.83, *p* ˂ 0.01), respectively (Figure 9). 

### 4.5. Difference among Observing and Simulating Crop Phenological Respond to Temperature

In Equation (1), variable a_nt_ is regression coefficient which shows the respond of crop phenology to variety change, planting date and temperature. While in Equation (3), only effect of temperature on simulated crop phenology is reflected by variable c_nt_ regression coefficient. If the difference (a_nt_ − c_nt)_ between regression coefficients is showed by negative value, then it means that local farming community grown short duration varieties in last years. If difference is positive then it point out that local farmers changed to longer thermal time requirement cultivars during last decades. Paired t-test was applied to determine the significance difference between regression coefficients.

## 5. Conclusions

The warming trend caused the changes in the field-observed phenological stages and phases of the cotton crop in Punjab, Pakistan, for the duration of 1980 to 2015 at 15 cotton-growing locations. Observed phenological stages S, E, A, and M occurred earlier by an average of 5.35, 5.08, 2.87 and 1.12 days decade^−1^, respectively. Observed S, E, A, and M were negatively correlated with the air temperature by an average of −2.03, −1.93, −1.09 and −0.42 days °C^−1^, respectively. The negative influence of the warming trend was partially diminished by the adaptation of growing new evolved cultivars with higher heat accumulation or growing degree day requirements. Approximately one-third of the negative impact of the enhanced temperature on the phenology of cotton was compensated for with the help of evolving new cultivars with higher total growing degree day requirements. 

## Figures and Tables

**Table 1:** Average observed phenology of cotton in Punjab, Pakistan, during the period of 1980–2015.

Districts	Sowing DOY	EmergenceDOY	Anthesis ^a^ DOY	Maturity ^b^ DOY
Toba Tek Singh	119 ± 7.0	124 ± 6.4	190 ± 5.4	274 ± 9.3
Jhang	121 ± 6.2	126 ± 5.7	188 ± 3.5	281 ± 7.3
Okara	128 ± 5.7	133 ± 5.3	191 ± 7.2	273 ± 5.4
Sahiwal	123 ± 7.2	128 ± 6.9	182 ± 6.1	278 ± 8.3
Pakpattan	130 ± 5.2	135 ± 5.0	189 ± 5.9	270 ± 7.8
Multan	120 ± 4.3	125 ± 4.1	191 ± 7.2	281 ± 6.7
Khanewal	125 ± 5.0	130 ± 4.8	193 ± 8.4	276 ± 5.5
Vehari	128 ± 6.7	133 ± 6.2	197 ± 9.2	285 ± 7.2
Lodhran	125 ± 4.4	130 ± 4.1	189 ± 6.2	278 ± 8.1
Bahawalnagar	131 ± 3.8	136 ± 3.5	192 ± 8.1	272 ± 9.5
Bahawalpur	130 ± 5.1	135 ± 4.5	187 ± 4.9	282 ± 8.5
DG Khan	126 ± 4.2	131 ± 3.9	183 ± 5.8	276 ± 6.7
Rajanpur	122 ± 6.9	127 ± 6.3	190 ± 7.3	280 ± 7.6
Muzaffargarh	127 ± 5.2	132 ± 4.7	194 ± 6.1	276 ± 7.1
Rahim Yar Khan	123 ± 4.8	128 ± 3.9	197 ± 6.8	270 ± 8.6

^a^ 50% Anthesis; ^b^ 90% Physiological maturity; DOY = Day of year.

**Table 2:** Summary of observed and simulated phenology response to temperature for cotton in Punjab, Pakistan, for 1980–2015.

Phenology	No. neg. ^a^	No. pos. ^b^	No. sig. neg. ^c^	No. sig. pos. ^d^	Reg. Mean ^e^ (days °C^−1^)
**Cotton stages and phases (observed)**					
Sowing	15	0	13	0	−2.03
Emergence	15	0	13	0	−1.93
Anthesis	15	0	11	0	−1.09
Maturity	15	0	14	0	−0.42
Sowing-Anthesis	15	0	12	0	−0.94
Anthesis-Maturity	15	0	11	0	−0.67
Sowing-Maturity	15	0	13	0	−1.61
**Cotton phases (simulated)**					
Sowing-Anthesis	15	0	14	0	−1.42
Anthesis-Maturity	15	0	13	0	−1.06
Sowing-Maturity	15	0	14	0	−1.97

^a^ Number of locations with negative regression coefficients; ^b^ Number of locations with positive regression coefficients; ^c^ Number of locations with significant negative regression coefficients; ^d^ Number of locations with significant positive regression coefficients; ^e^ Mean of regression coefficients.

**Table 3:** Comparison of the responses of cotton phenology with average temperature using the observed and simulated data in Punjab, Pakistan, during 1980–2015.

Phenology (Phases)	Regression Coefficient ^a^ (days °C^−1^)		Difference between obs. and sim. Regression Correlations (Days °C^−1^)	*t*-Test (*p*-Value)
	Observed Data	Simulated Data		
Sowing-Anthesis	−0.94	−1.42	0.48	0.0012 **
Anthesis-Maturity	−0.67	−1.06	0.39	0.0053 **
Sowing-Maturity	−1.61	−1.97	0.36	0.0021 **

^a^ Mean of regression coefficients; Obs. = observed; Sim. = simulated; ** Significant at the 0.01 probability level.

**Table 4:** Cotton cultivars in all locations during 1980–2015.

Sr. No.	Site Name	Cultivars
1	Toba Tek Singh	CIM-506a, FH-901e, FDH-228b, CYTO-124, BH-3297, B-803, MNH-552, IR-1524
2	Jhang	CIM-499a, CIM-446e, MS-240, Sitara-005, SLH-8, FH-685, BH-100a, NS-141
3	Okara	CIM-534c, CIM-554e, IR-1274, MNH-998, FH-901, CIM-110, CIM-435, CIM-602
4	Sahiwal	NIAB-111c, CIM-473d, GN-1532, CEMB-66, B-896, CIM-70, B-622, FVH-55
5	Pakpattan	BH-160e, MNH-786a, CIM-109, NIBGE-901, AGC-777, NIBGE-6, FS-631, CIM-240
6	Multan	IR-3701, CIM-240, CIM-1100, NIBGE-2d, AGC-999, CIM-109, B-820, BH-118
7	Khanewal	MG-6, NIAB-846d, CIM-707, Sitara-12, NIAB-2008e, S-12, MNH-554, FVH-57
8	Vehari	Sitara-008, NIAB-777d, CIM-600, FH-142, TCD-3, CIM-482a, MNH-93, VH-259
9	Lodhran	FH-113, CRSM-38d, TS-103, CYTO-177, FH-628b, FH-87, FH-657, MNH-516
10	Bahawalnagar	Neelam-121c, AH-151d, Tarzan-3, B-842, CIM-465c, RH-500, SLS-1, Tarzan-1
11	Bahawalpur	Ali Akbar-802, S-14 , MNH-786a, Leader-1, FDH-170b, NIAB-78, B-821, NIAB-26
12	DG Khan	IR-1524, NIBGE-1e, VH-305, FH-113, TSR-2375, Karishma, CIM-467, VH-137
13	Rajanpur	GN-2085, NIAB-846e, IUB-13, MS-240, MNH-465, BH-95, FH-649, MNH-536
14	Muzaffargarh	CIM-496c, Desi Ravib, FH-Lalazar, 149-F, S-12, FH-629, MVH-518, VS-135
15	Rahim Yar Khan	Ali Akbar-703, FDH-170b, AC-134, MM-58, CIM-448a, B-803, FVH-49, NIAB-846

Sr. No. = Serial number; a = American cultivar; b = local cultivars; c = short duration; d = medium duration; e = heat tolerant.
